# Identification of a Qualitative Signature for the Diagnosis of Dementia With Lewy Bodies

**DOI:** 10.3389/fgene.2021.758103

**Published:** 2021-11-19

**Authors:** Shu Zhou, Qingchun Meng, Lingyu Li, Luo Hai, Zexuan Wang, Zhicheng Li, Yingli Sun

**Affiliations:** ^1^ Institute of Biomedical and Health Engineering, Shenzhen Institutes of Advanced Technology, Chinese Academy of Sciences, Shenzhen, China; ^2^ Central Laboratory, National Cancer Center/National Clinical Research Center for Cancer/Cancer Hospital & Shenzhen Hospital, Chinese Academy of Medical Sciences and Peking Union Medical College, Shenzhen, China

**Keywords:** dementia with Lewy bodies, microRNA, relative expression ordering, signature, feature selection

## Abstract

**Background and purpose:** Diagnosis of dementia with Lewy bodies (DLB) is highly challenging, primarily due to a lack of valid and reliable diagnostic tools. To date, there is no report of qualitative signature for the diagnosis of DLB. We aimed to develop a blood-based qualitative signature for differentiating DLB patients from healthy controls.

**Methods:** The GSE120584 dataset was downloaded from the public database Gene Expression Omnibus (GEO). We combined multiple methods to select features based on the within-sample relative expression orderings (REOs) of microRNA (miRNA) pairs. Specifically, we first quickly selected miRNA pairs related to DLB by identifying reversal stable miRNA pairs. Then, an optimal miRNA pair subset was extracted by random forest (RF) and support vector machine-recursive feature elimination (SVM-RFE) methods. Furthermore, we applied logistic regression (LR) and SVM to build several prediction models. The model performance was assessed using the receiver operating characteristic curve (ROC) analysis. Lastly, we conducted bioinformatics analyses to explore the molecular mechanisms of the discovered miRNAs.

**Results:** A qualitative signature consisted of 17 miRNA pairs and two clinical factors was identified for discriminating DLB patients from healthy controls. The signature is robust against experimental batch effects and applicable at the individual levels. The accuracies of the-signature-based models on the test set are 82.61 and 79.35%, respectively, indicating that the signature has acceptable discrimination performance. Moreover, bioinformatics analyses revealed that predicted target genes were enriched in 11 Go terms and 2 KEGG pathways. Moreover, five potential hub genes were found for DLB, including SRF, MAPK1, YWHAE, RPS6KA3, and KDM7A.

**Conclusion:** This study provided a blood-based qualitative signature with the potential to be used as an effective tool to improve the accuracy of DLB diagnosis.

## Introduction

Dementia with Lewy bodies (DLB) is the second most common cause of neurodegenerative dementia, accounting for up to 15–20% of dementia patients (Mueller et al., 2017; Arnaoutoglou et al., 2019). An accurate diagnosis of DLB is vital for its treatment. This is mainly because patients with DLB react badly to some traditional and commonly used antipsychotic medications, notable medications with anticholinergic or antidopaminergic actions (McKeith et al., 1992). According to DLB diagnostic criteria released by the DLB consortium (2017 version), the diagnostic method of DLB in clinical practice is primarily based on clinical features, imaging parameters, and electrophysiological markers (McKeith et al., 2017). A highly suspected case of DLB is diagnosed when two or more of the core clinical features are present; or when only one core clinical feature is present, but with one or more indicative biomarkers. Although the consensus of diagnostic criteria is continuously developing, many patients with DLB remain undiagnosed or misdiagnosed (Hohl et al., 2000; Rizzo et al., 2018). Diagnosing DLB is highly challenging, mainly due to a lack of valuable and effective biomarkers, and its symptoms are similar to other dementia subtypes, such as Alzheimer’s disease (AD) (Noe et al., 2004). A valid and reliable diagnostic method for DLB is still in demand.

To date, some potential biomarkers for DLB diagnosis have been reported, such as α-synuclein (αSyn) (Spillantini et al., 1997), amyloid β1-42 (Aβ42) (Parnetti et al., 2008), and phosphorylated tau at threonine 181 (pTau) (Mollenhauer et al., 2006), etc. Among them, the biomarker αSyn, as a significant component of Lewy bodies, has been intensely investigated. Recently, more attention has been paid to discovering blood signatures because of their multiple advantages, including minimally invasive, readily available, and detectable. Several potential blood-based quantitative signatures have been discovered for DLB diagnosis. For example, Suzuki *et al.* developed a serum signature with four peptides (2,898, 4,052, 4,090, and 5002m/z) for discriminating DLB patients from AD patients and healthy controls (Suzuki et al., 2015). Another example is that Shigemizu *et al.* developed a serum signature consisting of 180 microRNAs (miRNAs) and two clinical factors (age and APOE ε4 genotype) to differentiate DLB patients from healthy controls (Shigemizu et al., 2019).

Although these reported quantitative signatures for DLB have achieved reasonable discriminatory capability, their application may be limited due to widespread batch effects. Therefore, it is of great significance to identify qualitative signatures that are insensitive to batch effects for DLB diagnosis. Some studies have indicated that REO-based qualitative signatures are robust against batch effects (Chen et al., 2017; Zhang et al., 2020). Moreover, several lines of evidence have revealed that miRNAs may be a contributing factor in neurodegeneration (Nelson et al., 2008; Junn and Mouradian, 2012). The miRNAs are small non-coding RNAs of 18–24 nucleotides in length (Mohr and Mott, 2015). They play crucial roles in many biological processes (Bushati and Cohen, 2007), such as proliferation (Corney et al., 2007; Johnnidis et al., 2008), apoptosis (Welch et al., 2007; Buscaglia and Li, 2011), differentiation (Esau et al., 2004; Makeyev et al., 2007). In our study, given the above background, we discovered a blood-based qualitative signature with the potential to be used for DLB diagnosis based on the REO patterns of miRNA pairs and two clinical factors.

## Materials and Methods

**FIGURE 1:**
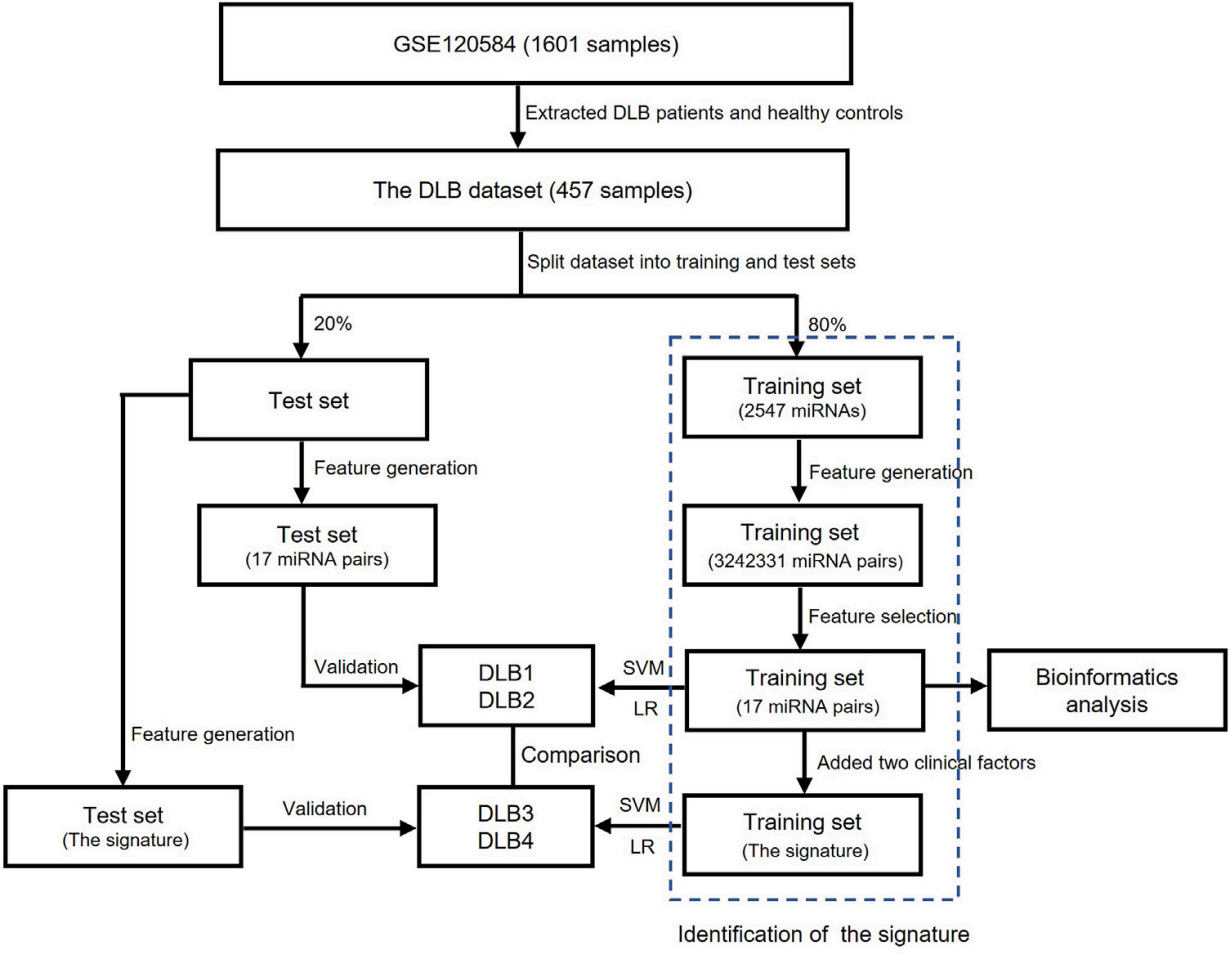
The flowchart of this study.

The flowchart of this study is shown in Figure 1. All feature selection and machine learning methods were implemented by python version 3.8.3. Dataset collection, preprocessing, and bioinformatics analysis were completed using R version 4.0.2 and web servers.

### Dataset Collection and Preprocessing

Firstly, datasets were retrieved from the GEO database using the keyword “dementia with Lewy bodies” of *Homo sapiens* (organisms). Then, the inclusion criteria were used as follows: 1) datasets contained DLB patients’ and healthy controls’ miRNA expression profiles; 2) samples were blood samples; and 3) information on age and APOE 4 genotype were provided. Finally, only one dataset GSE120584 was screened out and downloaded. The normalized miRNA expression matrix, platform set, annotation file, and corresponding clinical information were downloaded and parsed via the GEOquery package (Davis and Meltzer, 2007). The average expression value was taken as the miRNA expression value for multiple probes corresponding to a miRNA. The GSE120584 dataset contained 1021 AD patients, 91 vascular dementia (VaD) patients, 32 mild cognitive impairment (MCI) patients, 169 DLB patients, and 288 healthy controls. In our study, we aimed to develop a signature for differentiating DLB patients from healthy controls. Therefore, only the miRNA expression profiles and clinical information of 169 DLB patients and 288 healthy controls were extracted from the GSE120584 to construct a DLB dataset for analysis. Detailed information of the DLB dataset is listed in Supplementary Table S1. Then, we used the train_test_split function from the scikit-learn’s model_selection package to stratified and randomly select 20% samples from the DLB dataset to form an independent test set (34 DLB patients and 58 healthy controls). The random state for train-test-split was 16. The remaining samples were taken to construct a training set (135 DLB patients and 230 healthy controls). The training and test sets are listed in Supplementary Tables S2, S3, respectively. The distribution of samples in datasets is listed in Table 1. No significant correlation was observed between the training and test sets in clinical characteristics.

**TABLE 1:** The age, gender, and APOE ε4 genotype information in the training and test sets.

Characteristic	Type	Total	Training set	Test set	*p*-value
Age(year)	<65	50 (10.94%)	39 (10.68%)	11 (11.96%)	0.8710
	>=65	407 (89.06%)	326 (89.32%)	81 (88.04%)	
APOE^a^	0	356 (77.90%)	286 (78.36%)	70 (76.09%)	0.7427
	1–2	101 (22.10%)	79 (21.64%)	22 (23.91%)	
Gender	Female	238 (52.08%)	186 (50.96%)	52 (56.52%)	0.4022
	Male	219 (47.92%)	179 (49.04%)	40 (43.48%)	

a APOE, shows the average of the number of APOE ε4 allele genotypes. The *p*-value was calculated by the chi-squared test.

### Identification of a Qualitative Signature

In our study, three steps were performed to identify the qualitative signature for DLB, which were described as follows: 1) Feature generation. Given that the expression values of a miRNA pair (i, j) are denoted as Ei and Ej. The REO pattern of the miRNA pair is denoted as 1 (or 0 or −1 ) if Ei > Ej (or Ei = Ej or Ei < Ej). We calculated the values of the REO patterns for all miRNA pairs in each sample. The REO patterns of all miRNA pairs were used as new features for feature selection. 2,547 miRNAs constructed 3242331 miRNA pairs. 2) Feature selection. All feature selection methods were run on the training set. One miRNA pair was defined as a reversed stable miRNA pair when its REO pattern was identical in most control samples and was opposite in most patient samples. We first quickly identified 962 reversed stable miRNA pairs by setting the threshold at 60%. Then, the random forest (RF) was used to select 400 top-ranked important reversed stable miRNA pairs. The RF was implemented by the RandomForestClassifier function of the scikit-learn’s ensemble package. The random state was 16, and all other parameters were kept at default. Lastly, the support vector machine-recursive feature elimination (SVM-RFE) (Sanz et al., 2018) with stratified-3-fold cross-validation (SVM-RFE-CV) was applied to extract an optimal miRNA pair subset from 400 top-ranked important reversed stable miRNA pairs. SVM-RFE-CV was implemented by the RFECV function of yellowbrick’s model_selection package (Bengfort and Bilbro, 2019). Linear SVM was used as the base classifier. The penalty parameter of the error term is set to 1. All other parameters were kept at default. 3) The signature construction. According to the reference (Shigemizu et al., 2019), two clinical factors, age, and APOE ε4 genotype, may help to differentiate DLB patients from healthy controls. Therefore, we constructed the qualitative signature by combining the optimal miRNA pair subset and two suggested clinical factors. The numerical values of age were mapped to three classes (−1, 0, and 1) according to the thresholds at 70 and 80.

### Prediction Models’ Construction

Two commonly used machine learning methods, logistic regression (LR) (Sperandei, 2014) and SVM (Noble, 2006), were employed to build prediction models for discriminating DLB patients from healthy controls. Detailed principles of LR and SVM have been provided in previous papers (Noble, 2006; Sperandei, 2014). Thus, we only introduced the construction of our models in detail here. After identifying the optimal miRNA pair subset (17 miRNA pairs), we constructed new training and test sets by calculating the REO pattern’s values of these miRNA pairs. For comparison, we established four prediction models (DLB1, DLB2, DLB3, and DLB4) by LR and SVM based on 17 miRNAs pairs and the signature (17 miRNA pairs and two clinical factors), respectively. DLB1 and DLB2 were constructed with 17 miRNAs, while DLB3 and DLB4 were built with the signature. The training and test sets for four models are listed in Supplementary Tables S4, S5, S6, S7. SVM was implemented by the SVC function of scikit-learn’s SVM package. For all SVM models, the parameter probability was set to true. Given that the parameter gamma, penalty parameter of the error term, and kernel function are crucial for SVM models, we conducted a grid search to find their optimal values. All other parameters were kept at default. Moreover, LR was implemented by the LogisticRegression function of scikit-learn’s linear_model package. For all LR models, the parameters max_iter and penalty were set to 10000 and l2, respectively. The inverse regularization parameter was also tuned by grid search. All other parameters were kept at default. The grid search was implemented by the GridSearchCV function of scikit-learn’s model_selection package. Detailed information concerning search space and optimum values is summarized in Table 2. Here, all prediction models were validated using internal stratified-3-fold cross-validation and external test set techniques.

**TABLE 2:** Hyper-parameter search space considered for all models and optimized hyper-parameter values in the four models.

Model	Feature	Method	Hyper-parameter	Search space	Optimum value
DLB1	17 miRNA pairs	SVM	Penalty parameter of error term	[0.0002,0.002,0.2,2,20,200]	2
			Parameter gamma	[0.0002,0.002,0.2,2,20,200]	0.0002
			Kernel Function	[rbf, linear]	linear
DLB2		LR	Inverse regularization parameter C	[0.0001,0.001,0.01,0.1]	0.1
DLB3	The signature	SVM	Penalty parameter of error term	[0.0002,0.002,0.2,2,20,200]	20
			Parameter gamma	[0.0002,0.002,0.2,2,20,200]	0.002
			Kernel Function	[rbf, linear]	rbf
DLB4		LR	Inverse regularization parameter C	[0.0001,0.001,0.01,0.1]	0.1

### Models’ Performance Evaluation

Sensitivity (SE), specificity (SP), overall prediction accuracy, F1 score, and area under the receiver operating characteristic (ROC) curve (AUC) were calculated. ROC curves of the models were also plotted.

### Bioinformatics Analysis

We used the limma package to identify significantly dysregulated miRNAs of 21 miRNAs. Corrected *p*-value < 0.05 was considered significant. The miRWalk 3.0 online database (http://mirwalk.umm.uni-heidelberg.de/) and the mirDIP online database (http://ophid.utoronto.ca/mirDIP/) were used to predict target genes of these dysregulated miRNAs. TargetScan, miRDB, and miRTarBase datasets were incorporated into the miRwalk framework (Sticht et al., 2018). The cross-part between the genes identified by miRWalk and mirDIP were then extracted as target genes. Based on these target genes, the STRING 11.0 (https://string-db.org/cgi/input.pl), an online tool for retrieving interacting genes, was applied to construct a protein-protein interaction (PPI) network. The confidence score threshold was set to 0.9. Then, the CytoHubba (Chin et al., 2014), a well-known plugin of Cytoscape, was employed to identify hub genes. The eccentricity algorithm was selected, and all other plugin parameters were left at their default values. The five top-ranked genes were chosen as hub genes. Lastly, the ClusterProfiler (Yu et al., 2012), a widely used R package of Bioconductor, was used to perform gene ontology (GO) and Kyoto Encyclopedia of Genes and Genomes (KEGG) pathway enrichment analyses.

## Results

### Identification of the Qualitative Signature

**FIGURE 2:**
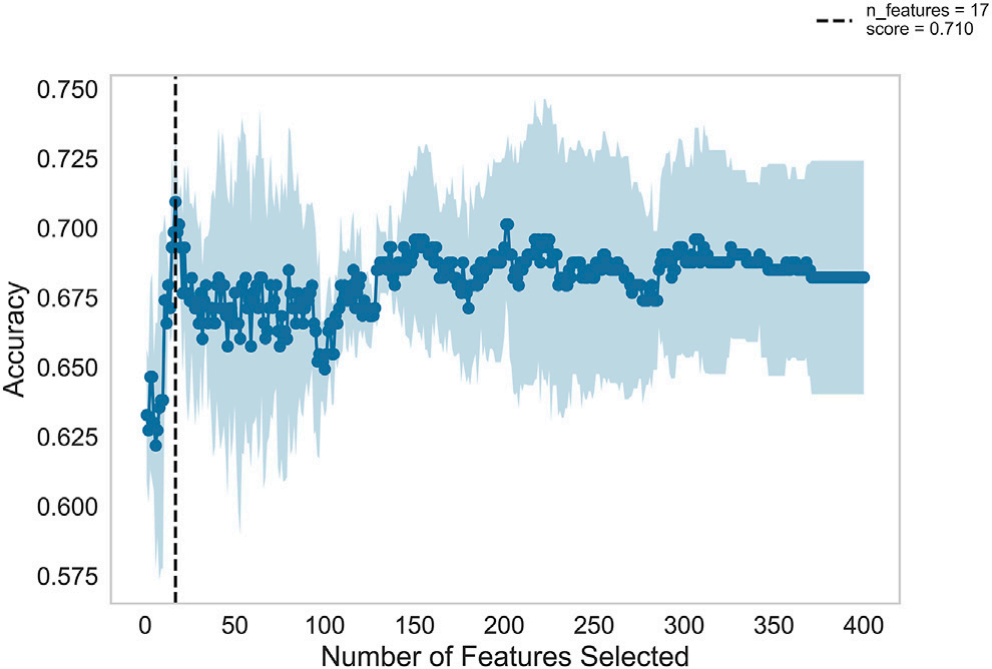
Feature recursive optimization. The dotted line shows the highest accuracy is achieved with 17 features. The horizontal axis is the number of feature selections, and the vertical axis is the prediction accuracy. Shadow areas represent the variability of cross-validation, with a standard deviation higher than and lower than the average accuracy score of curve drawing.

Figure 2 shows the automatic tuning of the number of features (miRNA pairs) selected by SVM-RFE-CV. From Figure 2, we observed that the highest accuracy, with 71%, came from 17 features. After 17 features, as non-information features are added to the model, the accuracy decreased gradually. After about 150 features, it entered a relatively steady state. Therefore, these 17 features were selected as the optimal feature subset, as shown in Table 3. These 17 miRNA pairs contain 21 miRNAs. Among them, there are nine significant dysregulated miRNAs (corrected *p*-value < 0.05). The expressions of hsa-miR-92a-2-5p and hsa-miR-6813-5p were downregulated in DLB patients with healthy controls. And the expressions of 7 miRNAs, including hsa-miR-5698, hsa-miR-5001-3p, hsa-miR-5195-3p, hsa-miR-4687-3p, hsa-miR-4793-5p, hsa-miR-1322, and hsa-miR-4756-3p, were upregulated. The remaining 12 miRNAs, including hsa-miR-3148, hsa-miR-335-3p, and hsa-miR-3677-3p, etc., were not significantly altered in DLB patients and healthy controls. More details are given in Supplementary Table S8.

**TABLE 3:** The information of the 17 miRNA pairs.

miRNA pair	miRNA i	miRNA j	miRNA pair	miRNA i	miRNA j
Pair 1	hsa-miR-4687-3p	hsa-miR-92a-2-5p	Pair 10	hsa-miR-5001-3p	hsa-miR-335-3p
Pair 2	hsa-miR-6813-5p	hsa-miR-5195-3p	Pair 11	hsa-miR-5001-3p	hsa-miR-140-3p
Pair 3	hsa-miR-1908-3p	hsa-miR-5698	Pair 12	hsa-miR-5001-3p	hsa-miR-3976
Pair 4	hsa-miR-5001-3p	hsa-miR-4756-3p	Pair 13	hsa-miR-6514-5p	hsa-miR-5001-3p
Pair 5	hsa-miR-5001-3p	hsa-miR-1322	Pair 14	hsa-miR-5001-3p	hsa-miR-561-3p
Pair 6	hsa-miR-5001-3p	hsa-miR-4477a	Pair 15	hsa-miR-5001-3p	hsa-miR-4793-5p
Pair 7	hsa-miR-5001-3p	hsa-miR-873-5p	Pair 16	hsa-miR-5001-3p	hsa-miR-875-3p
Pair 8	hsa-miR-5001-3p	hsa-miR-1253	Pair 17	hsa-miR-5001-3p	hsa-miR-3148
Pair 9	hsa-miR-5001-3p	hsa-miR-3677-3p			

### Prediction Models

**FIGURE 3:**
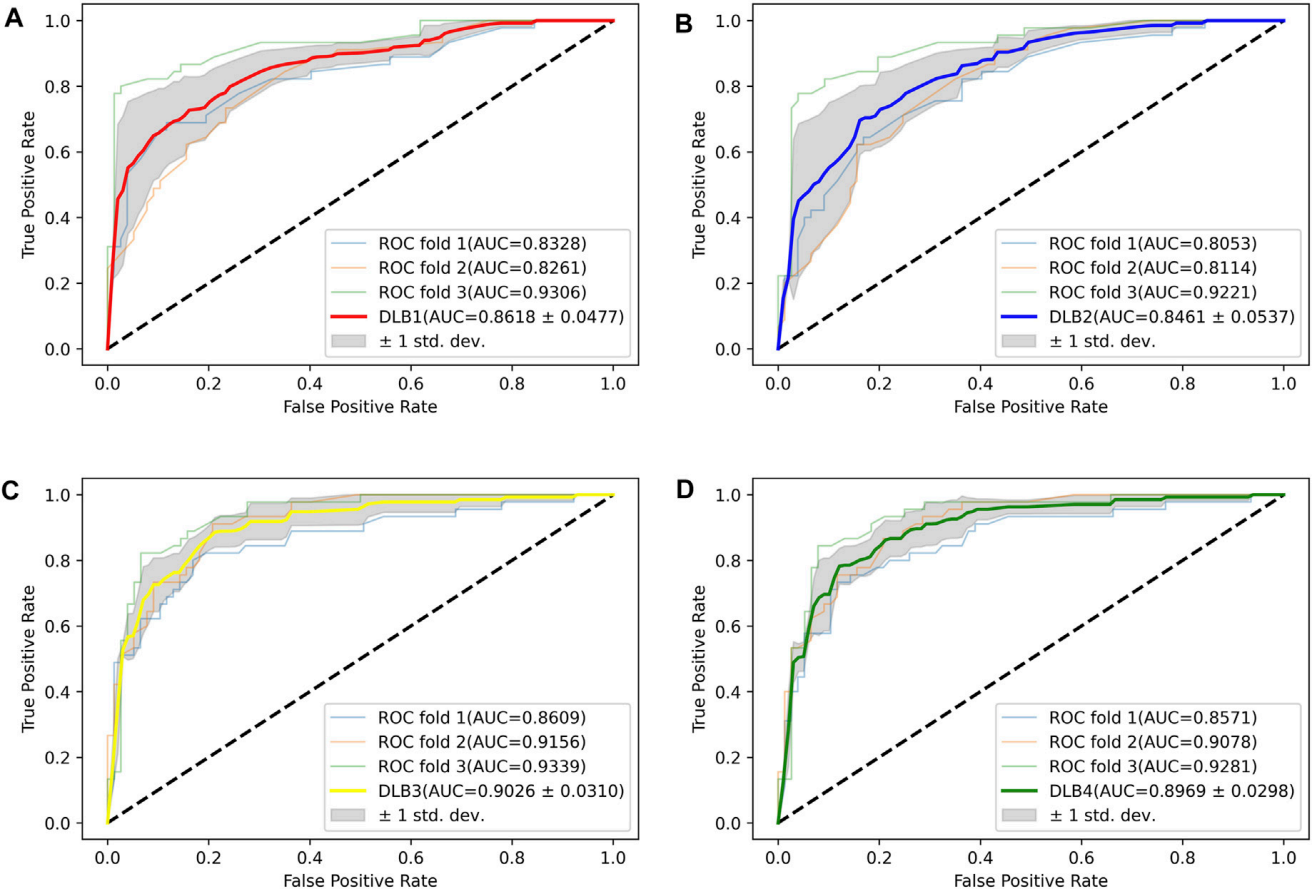
ROC curves for four models in the training set. **(A)** ROC curves of DLB1. **(B)** ROC curves of DLB2. **(C)** ROC curves of DLB3. **(D)** ROC curves of DLB4.

Based on 17 miRNA pairs and the signature, we built four models by SVM and LR, namely DLB1, DLB2, DLB3, and DLB4, respectively. These established models were firstly evaluated by the stratified-3-fold cross-validation method. The cross-validation results of the four models are shown in Table 4; Figure 3. From Table 4; Figure 3, we found that the DLB3 had the highest average sensitivity, F1 score, accuracy, and ROC AUC in the four models. The average sensitivity, F1 score, accuracy, and ROC AUC of the DLB3 are 72.59, 84.13, 84.39, and 90.26%, respectively. In the stratified-3-fold cross-validation of the DLB3, the ROC AUCs of two validation sets were as high as 93.39 and 91.56%, and the other one achieved 86.09%. However, the DLB1 provided the highest average specificity of 92.63%. It was higher than 90.89% of the DLB2, 91.31% of the DLB3, and 90.45% of the DLB4. The difference in the average specificity of the four models was slight, no more than 3%. Although the DLB1 provided better prediction in terms of specificity, it had lower sensitivity, F1 score, accuracy, and ROC AUC than the DLB3. In general, the DLB3 showed the best performance in the training set, and the DLB4 was second. Meanwhile, we observed that, except specificity, DLB3 and DLB4 performed better than DLB1 and DLB2 in terms of the other four evaluation criteria.

**TABLE 4:** Prediction results of the stratified 3-fold cross validation of four prediction models.

Model	Feature	Method	Fold number	SE (%)	SP (%)	F1-score (%)	Accuracy (%)
DLB1	17 miRNA pairs	SVM	1	48.89	96.10	76.87	78.69
			2	62.22	84.42	75.90	76.23
			3	80.00	97.37	90.73	90.91
			Average value	63.70	92.63	81.17	81.94
			Standard deviation	12.74	5.83	6.77	6.42
DLB2	17 miRNA pairs	LR	1	42.22	93.51	72.25	74.59
			2	57.78	84.42	74.07	74.59
			3	77.78	94.74	88.24	88.43
			Average value	59.26	90.89	78.19	79.20
			Standard deviation	14.55	4.60	7.15	6.52
DLB3	The signature	SVM	1	66.67	89.61	80.76	81.15
			2	71.11	90.91	83.33	83.61
			3	80.00	93.42	88.31	88.43
			Average value	72.59	91.31	84.13	84.39
			Standard deviation	5.54	1.58	3.13	3.02
DLB4	The signature	LR	1	62.22	89.61	78.94	79.51
			2	68.89	88.31	80.89	81.15
			3	77.78	93.42	87.44	87.60
			Average value	69.63	90.45	82.42	82.75
			Standard deviation	6.37	2.17	3.64	3.49

**FIGURE 4:**
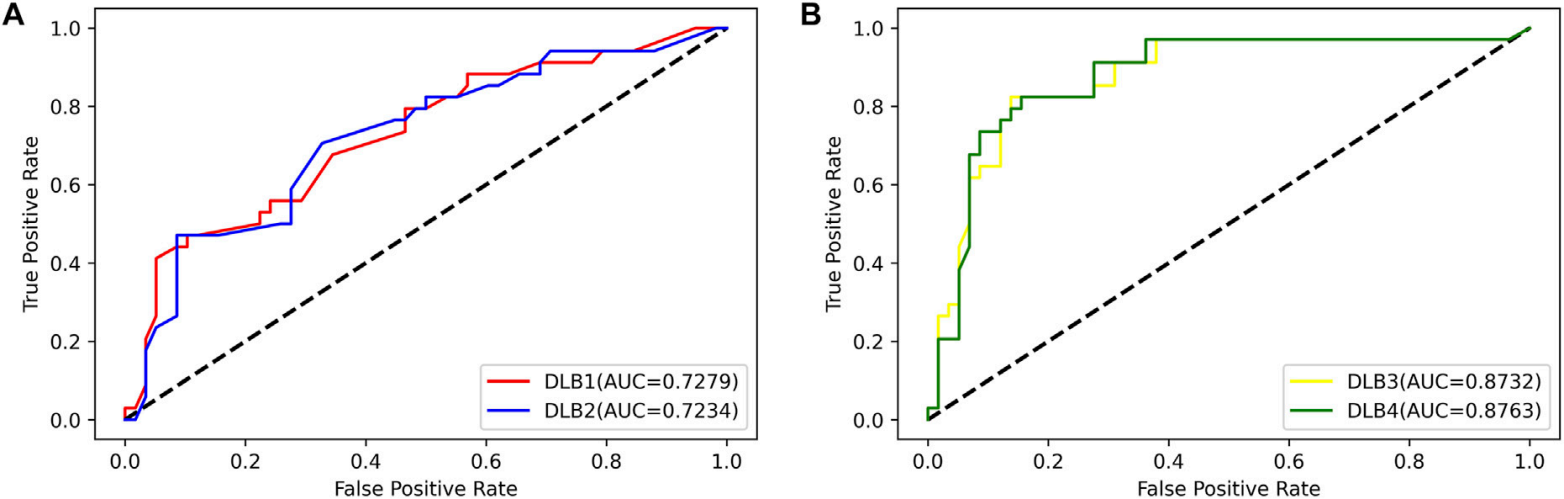
ROC curves for four models in the test set. **(A)** ROC curves of 17-miRNA-pairs-based models (DLB1 and DLB2). **(B)** ROC curves of the-signature-based models (DLB3 and DLB4).

Then, an independent test set was used to evaluate the predictability of these four models. Prediction results for the test set are shown in Table 5; Figure 4. Similar to the prediction results of the training set, DLB3 and DLB4 performed better than DLB1 and DLB2 in terms of sensitivity, F1 score, accuracy, and ROC AUC. Especially for sensitivity, compared to the DLB1 and DLB2, the sensitivities of the DLB3 and DLB4 improved by more than 20 and 17%, respectively. Sensitivity is of great importance within a diagnostic rule-out approach. The sensitivity, specificity, F1 score, accuracy, and ROC AUC of the DLB3 were 64.71, 87.93, 78.95, 79.35, and 87.32% in the test set. For the DLB4, they were 67.65, 91.38, 82.19, 82.61, and 87.63%, respectively. The DLB3 had the lowest specificity of 87.93%, while the other three models' specificity was 91.38%. In addition, unlike the training set results, the DLB4 was relatively more superior to the DLB3 in the test set under the evaluation of each evaluation criterion.

**TABLE 5:** The predicted results for four models in the test set.

Model	Feature	Method	SE (%)	SP (%)	F1-score (%)	Accuracy (%)
DLB1	17 miRNA pairs	SVM	44.12	91.38	71.94	73.91
DLB2		LR	47.06	91.38	73.31	75.00
DLB3	The signature	SVM	64.71	87.93	78.95	79.35
DLB4		LR	67.65	91.38	82.19	82.61

Overall, among these four models, the DLB3, which was developed by SVM based on the signature, outperforms other models in the training set. However, the DLB4 constructed by LR based on the signature provides the best prediction in the independent test set. Comparatively speaking, it is more important to classify external samples outside of the training set correctly. Therefore, in our study, the DLB4 is suggested to discriminate DLB patients from healthy controls. Meanwhile, we noted that the signature-based models perform better than 17-miRNA-pairs-based models in the training and test sets. These results indicate that integrating the clinical factors (age and APOE ε4 genotype) and 17 miRNA pairs improves the prediction performance.

### Bioinformatics Analysis

**FIGURE 5:**
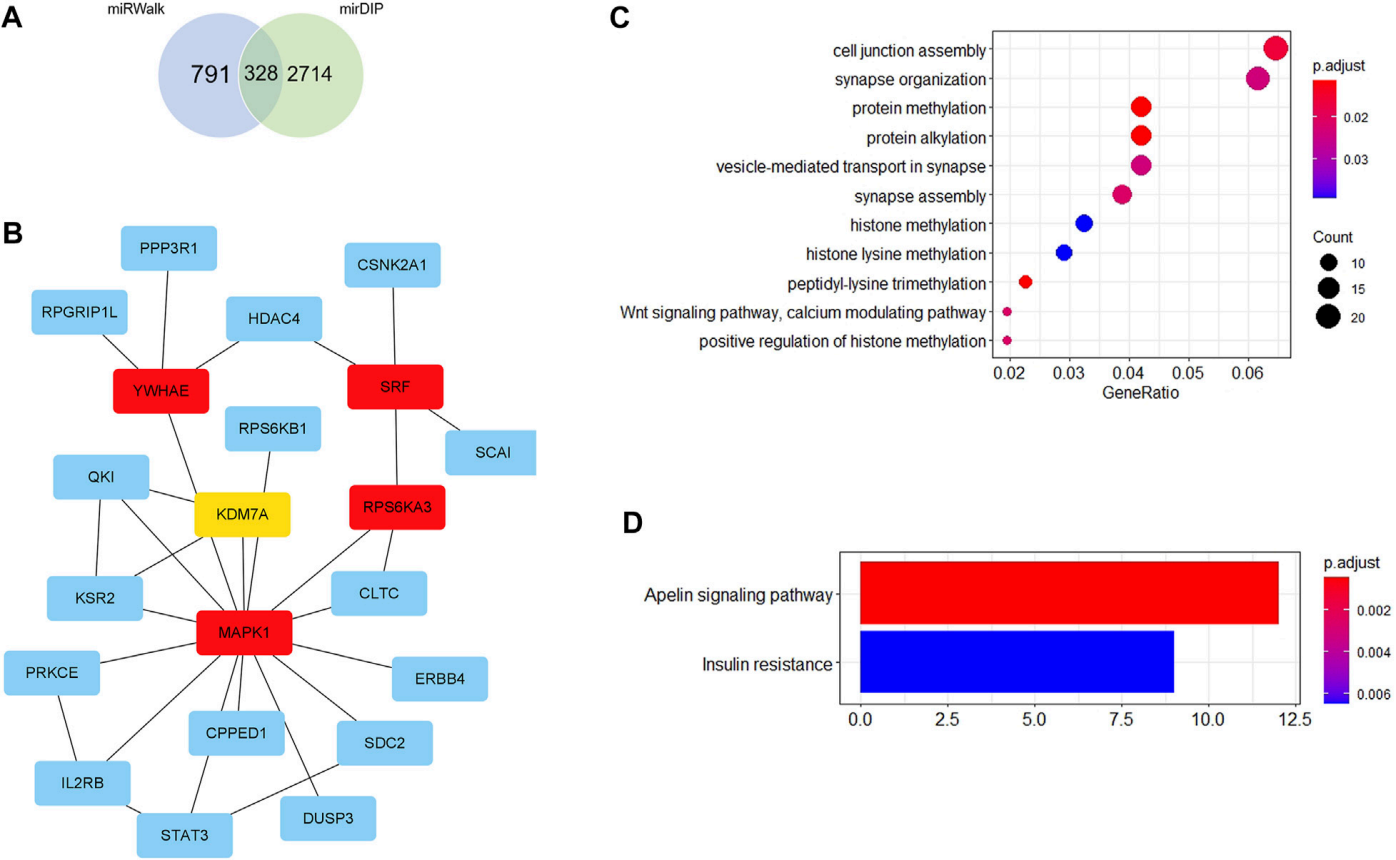
Bioinformatics analysis. **(A)** Venn diagram shows the overlapped target genes of 9 significant dysregulated miRNAs predicted by miRWalk and mirDIP. **(B)** Five hub genes with the most substantial interactions according to the calculated results by eccentricity method. The darker the color of nodes, the higher the score. **(C)** Bubble chart of 11 GO terms. **(D)** Bar chart of 2 KEGG pathways.

Firstly, 328 genes were predicted as target genes of these nine miRNAs by miRWalk and mirDIP (Figure 5A). Detailed information on these target genes is provided in Supplementary Table S9. A PPI network was established with 107 nodes and 173 edges by the STRING database (Supplementary Figure S1). The hub genes selected from the PPI network are shown in Figure 5B. The five highest-scored genes, including SRF, MAPK1, YWHAE, RPS6KA3, and KDM7A, were chosen according to the eccentricity scores. GO analysis revealed that 328 target genes were enriched in 11 terms, including cell junction assembly, synapse organization, protein methylation, protein alkylation, etc., as shown in Figure 5C. KEGG pathway analysis indicated that they were enriched in 2 pathways, including the apelin signalling pathway and insulin resistance (Figure 5D). More detailed GO and KEGG enrichment analyses are listed in Supplementary Tables S10, S11.

## Discussion

The object of this study is to identify a qualitative signature for DLB diagnosis. We conducted an analytical study of serum miRNA profiling and clinical information of 169 DLB patients and 288 healthy controls. The significant findings of the study were: 1) a qualitative signature that consisted of 17 miRNA pairs and two clinical factors was identified for the diagnosis of DLB; 2) Based on the signature, prediction models were established by LR and SVM. Among them, the DLB4 model performed the best, which offered an accuracy of 82.61% for the test set; 3) Five potential hub genes were discovered for DLB.

The main differences between our analysis and previous studies are exhibited in two aspects. On the one hand, as far as we know, a few quantitative signatures and no qualitative signatures have been reported for the diagnosis of DLB. This study discovered a blood qualitative signature consisted of 17 gene pairs and two clinical factors based on the REO pattern of the miRNA pair. The signature shows favorable discrimination capability, and it is robust and applicable to individual analysis. This is mainly because many biological and technical noise presented in the raw data is absorbed by the use of discrete classes (REO pattern). Several advantages of REO-based signatures have been demonstrated in numerous previous studies (Yan et al., 2018; Chen et al., 2020). For example, REO-based signatures are suitable for cross-platform measurements and comparisons because they are insensitive to sample normalization and experimental batch effects. Moreover, they could avoid bias in PCR micro-amplification, making them more feasible and convenient for clinical application.

On the other hand, we conducted a comprehensive bioinformatics analysis of potential target genes of nine dysregulated miRNAs of 21 miRNAs. Five hub genes were identified, including SRF, MAPK1, YWHAE, RPS6KA3, and KDM7A. Few, almost none of the studies so far have reported an association of them with DLB, but some evidence has implicated that they may play critical roles in other dementia subtypes. For example, SRF/MYOCD are suggested as novel targets for AD (Chow et al., 2007). They function as a transcriptional switch in the Aβ cerebrovascular clearance and progression of AD (Bell et al., 2009). To explore the molecular mechanism of these target genes, we analyzed their potential biological function and pathways. We found that they were enriched in 11 GO terms and 2 KEGG pathways. Most GO terms are related to synapse and protein methylation. Moreover, studies have reported that the apelin signalling pathway plays a vital role in neuroprotection (Cheng et al., 2012), and insulin resistance is associated with neurodegeneration (Suzanne, 2017).

Several limitations need to be acknowledged and addressed for this study. Firstly, to use these miRNA pairs as biomarkers, multicenter prospective studies will be required to evaluate the accuracy of DLB diagnosis. Secondly, some clinical characteristics associated with dementia were not analyzed in this study due to insufficient clinical information of these samples, such as hypertension, dyslipidemia, and diabetes. Thirdly, more basic studies will be required to study the possibility of these miRNAs being developed as biomarkers for DLB diagnosis. Lastly, we will focus on discovering signatures for differentiating DLB patients from other dementias in the future.

Overall, a blood qualitative signature consisted of 17 miRNAs and two clinical factors was identified to distinguish DLB patients from healthy controls in this study. The signature is highly robust against batch effects, and it is suitable for individual clinical applications. It is expected that the signature discovered in our research can be used as an effective tool to improve the accuracy of the diagnosis of DLB. Moreover, these new hub genes found may be potential targets for the treatment of DLB. More future studies will be required to explore the possibility of these hub genes being developed as targets in DLB.

## Data Availability

Publicly available datasets were analyzed in this study. This data can be found here: https://www.ncbi.nlm.nih.gov/geo/query/acc.cgi?acc=GSE120584&lt;/b&gt;.
